# Prophylactic Ketamine Treatment Promotes Resilience to Chronic Stress and Accelerates Recovery: Correlation with Changes in Synaptic Plasticity in the CA3 Subregion of the Hippocampus

**DOI:** 10.3390/ijms20071726

**Published:** 2019-04-08

**Authors:** Adam Krzystyniak, Ewa Baczynska, Marta Magnowska, Svitlana Antoniuk, Matylda Roszkowska, Monika Zareba-Koziol, Nirmal Das, Subhadip Basu, Michal Pikula, Jakub Wlodarczyk

**Affiliations:** 1 The Nencki Institute of Experimental Biology, Polish Academy of Sciences, 3 Pasteur Street, 02-093 Warsaw, Poland; e.baczynska@nencki.gov.pl (E.B.); m.pyskaty@nencki.gov.pl (M.M.); s.antoniuk@nencki.gov.pl (S.A.); m.babraj@nencki.gov.pl (M.R.); m.zareba-koziol@nencki.gov.pl (M.Z.-K.); 2 The Institute of Physical Chemistry, Polish Academy of Sciences, Kasprzaka Street 44/52, 01-224 Warsaw, Poland; 3 Cellular Neurophysiology, Centre of Physiology, Hannover Medical School, Carl-Neuberg-Str. 1, 30625 Hannover, Germany; 4 Department of Computer Science and Engineering, Jadvapur University, Kolkata 700032, India; das.nirmaljis@gmail.com (N.D.); subhadip@cse.jdvu.ac.in (S.B.); 5 Laboratory of Tissue Engineering and Regenerative Medicine, Department of Embryology, Medical University of Gdansk, 80-211 Gdansk, Poland; michal.pikula@gumed.edu.pl

**Keywords:** ketamine, depression, prophylactic antidepressant effect, structural plasticity, dendritic spines, stress recovery, CUS, chronic stress, CA3, sucrose preference

## Abstract

Ketamine is an *N*-methyl-d-aspartate receptor antagonist that has gained wide attention as a potent antidepressant. It has also been recently reported to have prophylactic effects in animal models of depression and anxiety. Alterations of neuroplasticity in different brain regions; such as the hippocampus; prefrontal cortex; and amygdala; are a hallmark of stress-related disorders; and such changes may endure beyond the treatment of symptoms. The present study investigated whether a prophylactic injection of ketamine has effects on structural plasticity in the brain in mice that are subjected to chronic unpredictable stress followed by an 8-day recovery period. Ketamine administration (3 mg/kg body weight) 1 h before stress exposure increased the number of resilient animals immediately after the cessation of stress exposure and positively influenced the recovery of susceptible animals to hedonic deficits. At the end of the recovery period; ketamine-treated animals exhibited significant differences in dendritic spine density and dendritic spine morphology in brain regions associated with depression compared with saline-treated animals. These results confirm previous findings of the prophylactic effects of ketamine and provide further evidence of an association between the antidepressant-like effect of ketamine and alterations of structural plasticity in the brain

## 1. Introduction

Major depressive disorder (MDD) is one of the most common psychiatric diseases, with a lifetime prevalence of up to 16.9% in the United States. It is associated with despair and increases in anxiety and anhedonia [1,2]. Chronic as well as acute severe stress may precipitate or exacerbate depression and multiple studies have reported elevated stress hormones levels in depressed patients and in animal models of depression [3,4] In fact, rodents repeatedly infused with corticosterone developed depression-like phenotype [5] and approximately 50–70% of patients with endogenous Cushing syndrome are diagnosed with major depressive disorder [6] which indicates causative relation between stress and the disease. Still the underlying mechanism of this disease is not fully understood. Neuronal plasticity appears to be particularly affected in depressed patients and animal models of stress-related disorders. A smaller volume of several brain structures, notably subregions of the prefrontal cortex (PFC) and hippocampus, lower dendritic spine density, and alterations of dendritic spine morphology are hallmarks of MDD [7,8]. The extent of atrophy is correlated with the duration and severity of symptoms, and such pathogenic changes may persist even after recovery from depression [9,10,11].

Standard antidepressant therapy that is monoamine-based has very limited efficacy mostly because of extensive effective times [11,12,13]. The noncompetitive glutamate *N*-methyl-d-aspartate (NMDA) receptor antagonist, ketamine has been shown to produce long-lasting (up to 1 week) antidepressant effects within hours after a single injection of a subanesthetic dose. These effects have been documented in both clinical settings and animal models of depression [14,15]. Interestingly, some recent animal studies have shown that ketamine also has prophylactic effects when administered before the beginning of chronic stress exposure. Mice that received ketamine exhibited a decrease in immobility time in the forced swim test (FST) and better performance in the novelty-suppressed feeding test and were protected from depressive-like behavior in the learned helplessness paradigm. These effects, however, were dose-dependent and varied between different animal models. Moreover, the time between the ketamine injection and beginning of the stress procedure was shown to be a critical factor for the observed outcomes [16,17,18,19,20].

The present study investigated whether prophylactic ketamine administration affects synaptic plasticity in the long-term and protects the brain from the loss of synaptic connections after stress exposure and the recovery period. Ketamine administration before social defeat stress may alter behavior in mice and thus possibly decrease its efficacy. Thus, we used the chronic unpredictable stress (CUS) paradigm, which includes social defeat stress among other stressors and may minimize possible behavioral bias [19]. Using the CUS procedure, we were able to distinguish between stress-susceptible and stress-resilient animals based on anhedonia criteria. Our results showed that ketamine administration (3 mg/kg body weight) 1 h before the CUS procedure caused significant changes in dendritic spine density and morphology in brain regions associated with depression, including the prefrontal cortex and subregions of the hippocampus 3 weeks after administration. Dendritic spine density significantly differed in the CA3 subregion of the hippocampus between ketamine-treated anhedonic animals and their saline-treated counterparts, thus confirming previous observations of the role of CA3 in the prophylactic effect of ketamine [16].

Sucrose preference is a well-accepted parameter for measuring anhedonic behavior in mice [21]. In the present study, based on the sucrose preference test, there were more resilient animals in the ketamine-treated group than in the saline-treated group immediately post-stress. Interestingly, sucrose preference in ketamine-treated anhedonic animals during the recovery period returned to the levels of resilient animals significantly faster compared with the saline-treated group.

## 2. Results

### 2.1. Ketamine Treatment before the Chronic Unpredictable Stress Procedure Altered Sucrose Preference in C57BL6J Mice

**Figure 1:**
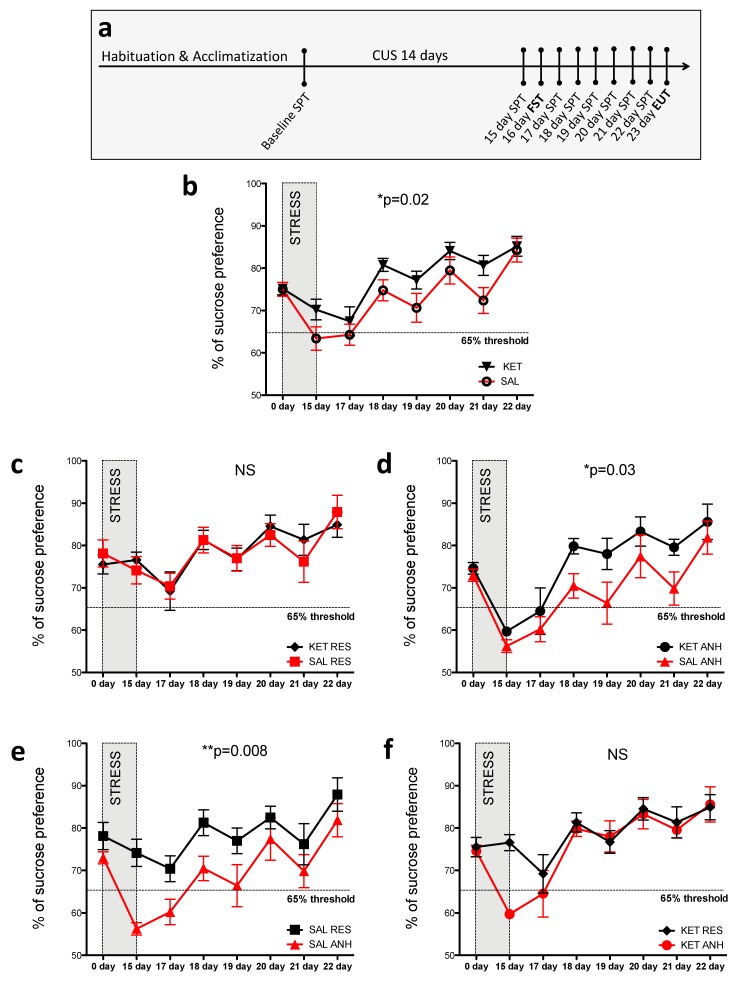
Ketamine treatment before the modified CUS procedure altered the sucrose preference profile in C57BL6J mice. (**a**) Schematic representation of the experimental procedure. After the acclimatization and habituation period, the mice were subjected to the 14-day CUS procedure, followed by 8 days of the daily sucrose preference test, with the exception of day 16 (i.e., 2 days after the end of CUS when the force swim test [FST] was performed). (**b**–**f**) Sucrose preference profiles in all of the mice (**b**), resilient mice (**c**), anhedonic mice (**d**), saline-treated mice (**e**), and ketamine-treated mice (**f**). The data are expressed as mean ± SEM. * *p* < 0.05, ** *p* < 0.01 (two-way ANOVA). Numbers of animals after CUS exposure: *n* = 16 ketamine-treated mice (six anhedonic mice and ten resilient mice), *n* = 15 saline-treated mice (nine anhedonic mice and six resilient mice).

In the present study, we tested whether a single injection of low-dose ketamine (3 mg/kg body weight) before the CUS procedure influences the development of a depressive phenotype and whether such ketamine treatment affects post-stress recovery. We measured sucrose preference in mice daily for 8 consecutive days post-stress, with the exception of day 2 when the FST was performed. The experimental design is outlined in Figure 1a. This modified CUS protocol is able to discern susceptible (anhedonic) and resilient (nonanhedonic) animals. Anhedonic animals were considered to exhibit <65% preference for sucrose over water in the sucrose preference test 24 h after the stress procedure. In the present study, we identified more anhedonic mice among saline-treated animals (nine anhedonic mice; i.e., 60% of all saline-treated mice, *n* = 15) compared with the ketamine-treated group (six anhedonic mice; i.e., 37% of all ketamine-treated mice, *n* = 16; Figure 1b).

According to a previous study, the recovery from hedonic deficits should occur after approximately 5 days post-stress [20]. Ketamine treatment before stress exposure significantly improved the sucrose preference profile throughout the course of the 8-day recovery period compared with saline treatment (*p* = 0.022; Figure 1b). When we examined the profile of sucrose preference in different subgroups of stressed animals throughout the recovery period, we found that ketamine influenced the mean sucrose preference in only anhedonic animals and not resilient animals. No significant difference was found in the datasets that represented changes in sucrose preference in resilient animals that received either ketamine or saline before the stress procedure (Figure 1c), whereas these datasets differed significantly between anhedonic animals (*p* = 0.036; Figure 1d). Interestingly, when we examined the difference between anhedonic and resilient animals within treatment groups (saline or ketamine), ketamine improved the recovery of sucrose preference to levels that were observed in resilient animals (Figure 1f). In contrast, saline-treated anhedonic animals exhibited significant differences in sucrose preference compared with their resilient counterparts throughout the recovery period (*p* = 0.008; Figure 1e). No significant difference was observed in the FST results between the ketamine- and saline-treated stress groups (data not shown). However, the efficiency of stress protocol was confirmed by an increase in immobility time (control, stress-naive mice, 147.90 ± 5.23 *n* = 14; CUS stressed mice, 174.70 ± 5.81 *n* = 31; *p* = 0.006) and body weight loss (control, stress-naive mice, 105.10 % of initial weight ± 0.76 *n* = 14; CUS stressed mice, 96.84 % of initial weight ± 1.11 *n* = 31; *p* < 0.0001) in the stressed animals compared with controls.

### 2.2. Ketamine Treatment before the Chronic Unpredictable Stress Procedure Affected the Density and Morphology of Dendritic Spines after the 8-day Recovery Period

**Figure 2:**
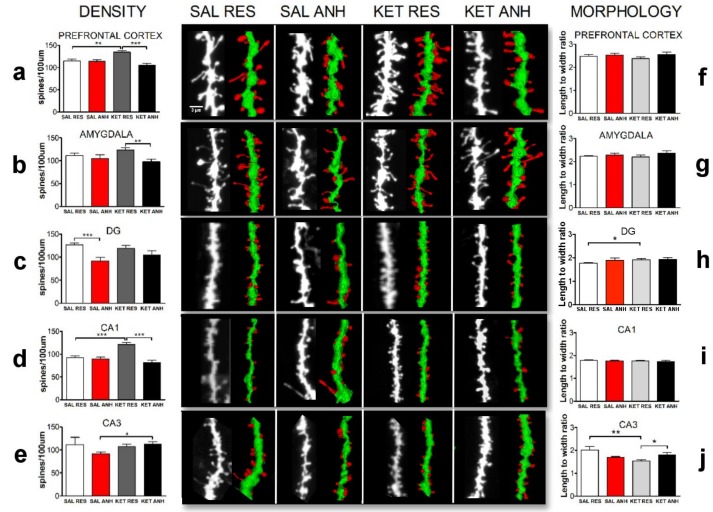
Ketamine treatment before the CUS procedure affected the density and morphology of dendritic spines on day 8 of the recovery period. The figure shows the density (**a**–**e**) and morphology (**f**–**j**) of dendritic spines in prefrontal cortex, amygdala, DG, CA1 and CA3 subregions of hippocampus of mice that were injected before CUS procedure with saline (SAL) or ketamine (KET) and followed CUS protocol leading to manifestation of anhedonic (ANH) or resilient (RES) behavior. The data are expressed as mean ± SEM. * *p* < 0.05, ** *p* < 0.01, *** *p* < 0.001. *n* ≥ 3 animals/group. Besides bar plots, the figure consists of representative confocal images together with 3D reconstructions of the depicted fragments of dendrites. The representative three-dimensional dendritic segment reconstructions were prepared using 3dSpAn software [22].

To further evaluate the impact of the prophylactic antidepressant-like effect of ketamine in a mouse model of depression, we focused on alterations of the structural plasticity of dendritic spines after the recovery period in brain areas that are associated with depression [2]. To study changes in plasticity, we analyzed dendritic spine density and morphology in the PFC, amygdala, and different subregions of the hippocampus (CA1, CA3, and dentate gyrus; Figure 2a–j). The analyses were performed based on the DiI staining of brain slices from at least six mice from the ketamine- and saline-treated groups. For this analysis, we selected at least three mice from each resilient and anhedonic group.

In ketamine-treated mice, spine densities were altered compared with saline-treated mice. A significant increase in spine density was observed in the PFC in ketamine-treated resilient mice (*n* = 15 cells, 134.65 ± 3.79 spines/100 µm) compared with ketamine-treated anhedonic mice (*n* = 12 cells, 104.90 ± 4.96 spines/100 µm) and saline-treated resilient mice (*n* = 15 cells, 114.60 ± 4.29 spines/100 µm; *p* = 0.0001 and *p* = 0.0016, respectively). No difference in spine density was found between anhedonic animals from both the saline- and ketamine-treated groups in the PFC (saline-treated anhedonic mice: *n* = 15 cells, 114.20 ± 3.67 spines/100 µm; ketamine-treated anhedonic mice: *n* = 12 cells, 104.90 ± 4.96 spines/100 µm; Figure 2a). When we looked at the hippocampus as a whole, we found significant differences within the ketamine-treated group (ketamine-treated resilient mice: *n* = 34 cells, 117.30 ± 3.46 spines/100 µm; ketamine-treated anhedonic mice: *n* = 20 cells, 98.04 ± 5.09 spines/100 µm; *p* = 0.0022) and saline-treated group (saline-treated resilient mice: *n* = 41 cells, 111.10 ± 4.08 spines/100 µm; saline-treated anhedonic mice: *n* = 27 cells, 90.86 ± 2.95 spines/100 µm; *p* = 0.0002). However, when we analyzed each subregion of the hippocampus separately, we found that ketamine differently altered spine density in the DG and CA1 and CA3 subregions. In the CA1 subregion, ketamine-treated resilient mice exhibited a significant increase in dendritic spine density compared with mice from the other groups (ketamine-treated resilient mice, *n* = 15 cells, 120.90 ± 4.97 spines/100 µm, vs. ketamine-treated anhedonic mice, *n* = 8 cells, 81.87 ± 4.66 spines/100 µm; *p* = 0.0001; saline-treated resilient mice, *n* = 16 cells, 92.64 ± 3.80 spines/100 µm, vs. ketamine-treated resilient mice, *n* = 11 cells, 120.90 ± 4.97 spines/100 µm, *p* < 0.0001; Figure 2d). The number of dendritic spines in the CA3 subregion in ketamine-treated anhedonic mice was significantly higher compared with saline-treated anhedonic mice (saline-treated anhedonic mice, *n* = 8 cells, 91.16 ± 4.54 spines/100 µm, vs. ketamine-treated anhedonic mice, *n* = 5 cells, 112.00 ± 6.3 spines/100 µm, *p* = 0.04; Figure 2e). Finally, in the DG, the spine density was significantly different within the saline-treated group (saline-treated resilient mice: *n* = 19 cells, 126.60 ± 4.08 spines/100 µm; saline-treated anhedonic mice: *n* = 8 cells, 91.98 ± 7.34 spines/100 µm; *p* = 0.0002) and not within the ketamine-treated group (ketamine-treated resilient mice: *n* = 13 cells, 118.80 ± 6.05 spines; ketamine-treated anhedonic mice: *n* = 8 cells, 104.80 ± 8.92 spines/100 µm; *p* > 0.05; Figure 2c). We did not observe significant differences in spine density in the amygdala in the saline-treated group (saline-treated resilient mice: *n* = 17 cells, 111.40 ± 5.72 spines/100 µm; saline-treated anhedonic mice: *n* = 13 cells, 104.80 ± 8.56 spines/100 µm; *p* > 0.05), but we observed significant differences in the ketamine-treated group (ketamine-treated resilient mice: *n* = 16 cells, 123.75 ± 4.72 spines/100 µm; ketamine-treated anhedonic mice: *n* = 8 cells, 97.87 ± 5.47 spines/100 µm; *p* < 0.0031; Figure 2b). Interestingly, we observed also a significant difference in dendritic spine morphology based on the scale-free parameter (spine length-to-head width ratio) in the CA3 subregion resulting spine maturation in ketamine resilient animals compared to saline-treated resilient mice and ketamine-treated anhedonic mice (saline-treated resilient mice, *n* = 12 cells, 2.01 ± 0.15 spines, vs. ketamine-treated resilient mice, *n* = 9 cells, 1.54 ± 0.04 spines, *p* = 0.005; ketamine-treated resilient mice, *n* = 9 cells, 1.54 ± 0.04 spines, vs. ketamine-treated anhedonic mice, *n* = 7 cells, 1.81 ± 0.09 spines, *p* = 0.011; Figure 2j) and in the DG subregion resulting elongation of spines in ketamine-treated resilient animals compared to their saline counterparts (saline-treated resilient mice, *n* = 25 cells, 1.77 ± 0.02 spines, vs. ketamine-treated resilient mice, *n* = 13 cells, 1.91 ± 0.05 spines, *p* = 0.02; Figure 2h).

## 3. Discussion

In the present study, we found that an injection of a subanesthetic dose of ketamine (3 mg/kg body weight) in mice before the modified CUS procedure was associated with pronounced behavioral and neurostructural effects that were detected after the 8-day recovery period. The present CUS paradigm allowed the discernment of anhedonic (susceptible) and resilient populations of mice. Such a distinction is important because not all individuals that are exposed to stress develop psychopathology [23,24], including both humans and other mammals (e.g., rodent species) that are used in biomedical research [25]. Notably, such variability in the stress response is evident even in inbred strains. In animal models, resilience is mediated by active neurobiological mechanisms that fail in anhedonic individuals [26,27]. Substantial evidence indicates that antidepressant therapy may be useful as a form of prophylaxis in depressed patients by increasing resilience [28,29]. Maintenance treatment with selective serotonin reuptake inhibitors (SSRIs) that extends beyond the management of acute depressive episodes has been shown to decrease the chance of recurrence in MDD patients. Nonetheless, no studies have evaluated the prophylactic effects of SSRIs in the healthy human population. Animal studies have provided some evidence that SSRI treatment before stress exposure exerts protective effects against anxiety and depressive behavior. This effect, however, depends on the specific stress paradigm and may occur as a result of chronic treatment (3 weeks) with the antidepressant that produces expected antidepressive effects rather than prophylaxis [30]. Prophylactic pharmacotherapy have been used in other stress-related disorders. Administration of morphine, propranolol or hydrocortisone before or within a short period after trauma decreased chance and severity of post-traumatic stress disorder (PTSD) [31]. Similarly, lithium and antiepileptic drugs were found to have the beneficial effect on bipolar disorder patients when used as prophylaxis [32]. To our knowledge, there is no data describing the prophylaxis of stress-related disorders with ketamine in clinical settings.

Ketamine is an NMDA receptor antagonist that has gained wide attention as a possible antidepressant. The antidepressant effect of a single injection of ketamine is not only rapid (i.e., detectable within 2 h after administration) but also long-lasting (up to 7 days) [33,34]. Ketamine has been shown to reverse behavioral symptoms of depression and act on structural and molecular changes in the brain [35]. Several studies have reported improvements in different parameters of depression in animal models and small human clinical trials following injections of subanesthetic doses of ketamine [14,15,36,37,38,39]. Recent studies have explored the prophylactic effects of ketamine. Brachman et al. [19] reported that a single injection of ketamine before social defeat stress, the learned helplessness paradigm, and chronic corticosterone administration led to significant antidepressant-like effects, such as decreases in immobility, social avoidance, and anxiety. The marked differences in effective doses of ketamine between different paradigms are particularly interesting, ranging from a relatively low dose (30 mg/kg body weight) to an anesthetic dose (90 mg/kg body weight) [19]. In a similar study that was performed by Donahue et al. [20], a comparable dose of ketamine (20 mg/kg body weight) that was administered before chronic social defeat stress did not significantly improve parameters of depressive-like behavior, but this treatment was effective when administered after the last social defeat session [20] (*n*.b., this latter experiment was not performed by Brachman et al. [19]. This suggests that choosing the optimal dose might be critical for obtaining the desired behavioral outcome. Dosing, together with other variables, could explain multiple discrepancies that are reported in the literature concerning the use of ketamine as an antidepressant agent [40,41,42,43]. Hedonic deficits that can occur as a result of chronic stress have been shown to be either reversed or unaffected by ketamine, depending on the dose and behavioral paradigm. Previous studies that observed the reversal of hedonic deficits used relatively low doses of ketamine [20,32,44]. To our knowledge, no studies have evaluated the prophylactic effects of ketamine on sucrose preference. In the present study, we administered 3 mg/kg body weight ketamine before the modified CUS paradigm. The ketamine-treated group exhibited a significant increase in average sucrose preference throughout the 8-day recovery period. When we looked at sucrose preference in anhedonic and resilient animals separately, we found no difference in average sucrose preference in these groups but rather a pronounced increase in the number of resilient animals immediately after cessation of the CUS procedure (15 day) in the ketamine-treated group. We further monitored sucrose preference in all of the animals throughout the 8-day recovery period to determine possible differences in the profile of recovery from hedonic deficits. Using our approach for monitoring recovery rate we found unexpectedly that the resilient groups had an almost identical recovery profile, whereas the anhedonic groups differed significantly. Anhedonic mice that were treated with ketamine before stress exposure exhibited more rapid recovery compared with saline-treated anhedonic mice. Notwithstanding these interesting findings, some caveats need to be considered. Average sucrose preference increased during the recovery period in all of the animals, but most of the anhedonic animals reached >65% sucrose preference (i.e., the threshold of anhedonia) very quickly. This was likely attributable to the fact that frequent sucrose preference testing (i.e., performed daily in the present study) may result in an increase in sucrose preference on its own, even in stress-naive animals [45]. Another interesting finding was the apparent oscillations in the sucrose preference test results during the recovery period. Each increase in sucrose preference was followed by a slight decrease what made the recovery profile step-wise rather than linear. This might reflect a type of desensitization cycle of reward circuits that may be more pronounced in anhedonic animals, which thus disrupted the perception of pleasure because of chronic stress. After 8 days of recovery when most of the animals reached the same level of sucrose preference, we evaluated whether the behavioral effects that we observed were reflected by changes in synaptic plasticity. We measured dendritic spine morphology and density in different brain regions that are associated with depression (i.e., the hippocampus, PFC, and amygdala). Structural alterations of those regions have been associated with different behavioral aspects of depression, such as memory impairment, a decrease in motivation, anxiety, and hedonic deficits [8]. Our results showed that the CUS procedure had a pronounced impact on dendritic spine density in the hippocampus even 8 days after stress exposure. This is consistent with previous studies that showed that the hippocampus is particularly prone to the detrimental effect of long-term stress, and a smaller volume of that structure may be considered a “scar” that remains after stress exposure. Furthermore, different subregions of the hippocampus are differentially affected by chronic stress, and anhedonic animals exhibit a decrease in dendritic spine density in the CA3 subregion and DG of the hippocampus immediately after the cessation of stress [46,47,48,49,50]. In the present study, the number of dendritic spines in the DG in saline-treated anhedonic mice after the 8-day recovery period was significantly less than in resilient animals, whereas spine density in the CA1 and CA3 subregions were not significantly different. This may suggest that those subregions but not DG recover at least partially after stress, and this recovery is correlated with the alleviation of hedonic deficits. This notion was underscored by the finding that ketamine-treated anhedonic mice exhibited a significant increase in spine density in the CA3 compared with their saline-treated counterparts. Structural plasticity is strongly affected by chronic stress, and a decrease in spine density in CA3 is associated with depressive behavior [51]. Recent studies showed that activation of the ventral CA3 is necessary for the prophylactic action of ketamine, and NMDA receptors in the CA3 subregion appear to play a critical role in the antidepressant action of the drug [16,38], which is consistent with the present results. Ketamine may induce long-lasting synaptogenesis in that structure, making it less prone to stress hormone-induced plasticity. Interestingly, spine densities in ketamine-treated resilient animals were significantly higher in the prefrontal cortex and CA1 compared with saline-treated mice. Since CA1 and PFC (infralimbic medial PFC) have been shown to be less affected in the murine model of depression [49], thus it is possible that resilient phenotype produces environment that protects from detrimental effects of stress in those regions. In a recent in vivo study a single ketamine injection induced a long-lasting increase in spine density in the PFC. This effect was driven by an elevation of the rate of spine formation and the stability of newly formed spines [52]. Perhaps neuroprotective resilience mechanisms act synergistically with ketamine action promoting synaptogenesis.

Chronic stress and acute stress not only influence dendritic spine density in different brain regions but also change their morphology [53]. Different types of dendritic spines can be categorized into three basic categories that are based on their shape (i.e., thin, mushroom, and stubby spines). Mushroom spines usually have a relatively large spine head relative to its base, whereas thin and stubby spines do not have distinct head formation. Mushroom spines are considered more mature spines that are associated with long-term memories and neuronal network stability [8]. Spine classifications are based on simple, arbitrary criteria and are prone to bias. To decrease the possibility of misinterpreting the prophylactic effect of ketamine on spine morphology, we calculated the spine length-to-head width ratio. In our previous studies, we found that this scale-free parameter is useful for quantifying synaptic plasticity [22,54,55]. Using this approach, we found that dendritic spine morphology was significantly altered only in the DG and CA3 subregion of the hippocampus at the end of the recovery period. Stress has been shown to selectively increase the number of thin and stubby spines in the PFC [56] and decrease the number of thin and stubby spines in different subregions of the hippocampus [57]. However, this effect was reversible after the recovery period [58]. In the present study, we found that ketamine treatment before stress exposure produced morphological changes in the pool of dendritic spines at the end of behavioral recovery period. Prophylactic ketamine administration appeared to affect the spine length-to-head width ratio in the group of resilient animals only. In these animals, spines in the DG were less mature, suggesting that this region underwent more plasticity and may be associated with adaptation to new conditions. In the CA3 in ketamine-treated resilient animals, we found a significantly lower spine length-to-head width ratio compared with their saline-treated counterparts and the ketamine-treated anhedonic group. A lower spine length-to-head width ratio indicates more mature synaptic connections and a more stable network. Determining the direction of changes in morphology is difficult because the effects that we observed may be attributable to an increase in spine formation or a decrease in spine elimination [59]. In stress-naive animals, ketamine facilitated spine formation, but it may act differently under conditions of stress, with sex-dependent effects [60]. Clarifying this issue will require in vivo brain imaging, but such investigations were beyond the scope of the present study.

We did not find significant differences in the FST results between ketamine- and saline-treated animals, but we observed an increase in immobility time in all of the stressed animals compared with controls, thus confirming the effectiveness of the stress procedure. Lack of prophylactic effect of low dose of ketamine in the FST compared to saline treated stressed animals was previously shown in the social defeat stress model [19].

In conclusion, we found that prophylactic ketamine treatment before CUS increased resilience to stress and accelerated recovery from depressive-like behavior. This effect of ketamine may be associated with changes in structural plasticity in brain regions that are implicated in depression, particularly the CA3 subregion of the hippocampus. Our data suggest that frequent monitoring of the recovery rate from depressive behavior in animal models of stress related disorders may be considered as another parameter in evaluating potential antidepressive therapies.

## 4. Materials and Methods

### 4.1. Animals and Housing Conditions

Male 3-month-old C57BL6J mice from Jackson Laboratory (Białystok, Poland) were individually housed under a reverse 12 h/12 h light/dark cycle (lights on at 8:00 PM) with food and water available ad libitum. Male CD1 mice were purchased from Charles Rivers (Sulzfeld, Germany). They were used as resident intruders in the social defeat stress procedure and kept in the same animal room. Male Wistar rats from Harlan (Warsaw, Poland) were used for the predator stress procedure. All of the animal experiments were approved by the 1st Warsaw Ethical Committee on animal research (permission no. 132/2016 from 14.12.2016).

### 4.2. Drug Administration

A single intraperitoneal injection of saline (0.9% sodium chloride) or (R,S)-ketamine hydrochloride (3 mg/kg body weight; Biowet Pulawy) was administered 1 h before the modified CUS procedure.

### 4.3. Experimental Design

To establish the mouse model of depression, we used a modification of the previously described CUS protocol and behavioral evaluation methods [59,61] under a reverse 12 h/12 h light/dark cycle. After 1 week of acclimatization and 1 week of habituation, the mice were weighed and tested for social behavior, and their baseline sucrose preference was recorded. They were then assigned to a control group (*n* = 12) and stress group (*n* = 32) housed in two separate rooms. Half of the mice from both the stress group and control group were intraperitoneally injected with either saline or ketamine (3 mg/kg body weight). The mice were injected 1 h before the first stress session. The CUS protocol consisted of three different stressors that were applied daily during the dark phase under red light in the following sequence: Restraint stress for 2 h, tail suspension for 40 min, and social defeat stress for 30 min, with an intersession interval of at least 4 h. During the light phase, the mice were exposed to a rat. Approximately 16 h after the last stressor, the mice underwent an 8-h sucrose preference test, followed by the forced swim test the next day. To stabilize glucocorticoid levels after the last stressor, the mice were left undisturbed overnight before beginning the sucrose preference test [62,63]. Sucrose preference was assessed daily in all of the mice for the next 6 consecutive days, for a total of seven sucrose preference tests during the 8-day recovery period. All of the mice were sacrificed 24 h after the last sucrose preference test. The experimental design is outlined in Figure 1a.

### 4.4. Mouse Model of Depression based on Modified Chronic Unpredictable Stress Protocol

The modified CUS procedure consisted of 2 weeks of stress exposure. The stressors were applied in a semi-random order during the dark and light phases of the light/dark cycle.

*Restraint stress*. The mice were placed inside a plastic tube (26 mm internal diameter) for 2 h during the dark phase of the light/dark cycle and kept in a dark experimental room.

*Tail suspension stress*. The mice were subjected to the tail suspension procedure by hanging them by their tails using adhesive tape for 40 min during the dark phase of the light/dark cycle. To prevent the mice from climbing their tails, plastic cylinders (0.5 cm × 4 cm) were placed at the base of their tails.

*Social Defeat Stress*. This procedure was performed during the dark phase under red light to observe mouse behavior. First, CD1 mice that attacked C57BL6J mice in less than 60 s without injuring them were selected for this procedure. During each 30-min social defeat session, these aggressive CD1 animals were placed in the home cages of C57BL6J mice in the stress group. During each session, the C57BL6J mice exhibited signs of social defeat stress, such as a flight response, submissive posture, and vocalization. If the mice in the stress group did not display signs of social defeat stress, then the CD1 mouse was changed to another CD1 mouse. In rare cases of physical harm that occurred between pairs of mice, aggressive CD1 individuals were immediately removed from the cage of the C57BL6J resident mice.

*Exposure to a Rat.* The mice were individually introduced into transparent, well-ventilated cylinders (15 cm × 8 cm) with food and some bedding. The cylinders were then placed for 12 h (08:00 PM–08:00 AM) into a rat’s home cage that contained a rat. For the rest of the day (08:00 AM–08:00 PM), the mice were housed in their home cages in the same experimental room.

### 4.5. Behavioral Tests

#### 4.5.1. Sucrose Preference Test

In this test, the mice were given free-choice access to 1% sucrose solution and water that were provided in identical bottles for 8 h. The percentage of sucrose preference was calculated as the following:

Sucrose Preference = (Weight_Sucrose solution_/[Weight_Sucrose solution_ + Weight_Water_]) × 100%

The consumption of water and sucrose solution was estimated simultaneously in the control and experimental groups by weighing the bottles. To eliminate possible bias from side preference, the positions of the bottles were changed after 4 h of the test. Twenty-four hours before the baseline sucrose preference test, 2.5% sucrose solution was given to all of the animals for 2 h to prevent possible effects of taste neophobia. The other conditions of the test were as previously described [45,59]. Sucrose preference <65% in mice in the stress group measured 24 h after cessation of the stress procedure was the criterion for “anhedonia.” This criterion was based on the fact that none of the control animals exhibited <65% sucrose preference. Stressed mice with sucrose preference >65% at the end of the experiment were defined as resilient. Anhedonic mice were previously shown to display depressive-like phenotype [64].

#### 4.5.2. Forced Swim Test

In this test, cylindrical glass containers (20 cm × 40 cm) were filled with warm water (~30 °C) to a depth of 15 cm. The test was conducted under red light during the dark phase of the light/dark cycle. Each mouse was placed in the water for 6 min in a single swim session. The latency to the first episode of floating (no body or head movements for more than 2 s) and the sum of floating time during the last 4 min were measured by visual scoring offline.

#### 4.5.3. DiI Staining of Brain Slices

To visualize changes in the shape of dendritic spines, 1,1′-dioctadecyl-3,3,3,3′-tetramethylindocarbocyanine perchlorate (DiI) staining was performed in brain sections from stressed. The mice were anesthetized and transcardially perfused with 1.5% paraformaldehyde. The brains were dissected and sliced using a vibratome. Slices (140 µm thick) that contained the different brain structures were allowed to recover for at least 1.5 h at room temperature. Random dendrite labeling was performed using 1.6 µm tungsten particles (Bio-Rad, Hercules, CA, USA) that were coated with propelled lipophilic fluorescent dye (DiI; Invitrogen) that was delivered to the cells by gene gun (Bio-Rad) bombardment. Images of dendrites in different brain regions (e.g., Prefrontal Cortex (without making distinction between subregions of the structure), Hippocampus (CA1, CA3 and Dentate Gyrus) and Amygdala (without making distinction between subregions of the structure)), were acquired under 561 nm fluorescent illumination using a confocal microscope (63x objective, 1.4 NA) at a pixel resolution of 1024 x 1024 with a 3.43 zoom, resulting in a 0.07 µm pixel size.

### 4.6. Morphometric Analysis of Dendritic Spines

The analysis of dendritic spine morphology and calculation of changes in spine parameters were performed as described previously [54,65]. The images that were acquired from the brain slices were processed using ImageJ software (National Institutes of Health, Bethesda, MD, USA) and analyzed semi-automatically using custom-written SpineMagick software (patent no. WO/2013/021001) and 3dSpAn software for three-dimensional dendritic segment reconstruction [30]. The analyzed dendritic spines belonged to secondary and ternary dendrites to reduce possible differences in spine morphology that are caused by the location of spines on dendrites with different ranks. Moreover, spontaneous changes in dendritic spine shape can obscure systematic effects because of the spontaneous intrinsic fluctuation of dendritic spine shape [66]. To minimize this effect in the analysis, we used a scale-free parameter of relative changes in the spine length-to-head width ratio, which reflects spine shape. The spine length was determined by measuring the curvilinear length along a fitted virtual skeleton of the spine. The fitting procedure was performed by looking for a curve along which integrated fluorescence was at a maximum. The head width was defined as the diameter of the largest spine section while excluding the bottom part of the spine (1/3 of the spine length adjacent to the dendrite). Dendritic segments of at least 3 animals per condition were morphologically analyzed resulting in 400–1700 spines. To determine spine density, approximately 800-8000 µm of dendritic length was analyzed per experimental group.

### 4.7. Statistical Analysis

Sucrose preference data was analyzed using two-way analysis of variance (ANOVA) followed by the Bonferroni post hoc test. For the statistical analysis of synaptic plasticity (density and morphology), we used the parametric unpaired Student’s t-test (equal variances in Bartlett’s test, and normal distribution in D’Agostino and Pearson omnibus normality test) or nonparametric t-test with Welch correction (unequal variances) or the Mann–Whitney test (lack of normality). Values of p < 0.05 were considered statistically significant. The analyses were performed using Prism 5 software (GraphPad, San Diego, CA, USA).

## Abbreviations

AMY	Amygdala
CUS	Chronic Unpredictable Stress
DG	Dentate Gyrus
FST	Forced Swimming Test
NMDA	*N*-methyl-d-aspartate
SPT	Sucrose Preference Test
PFC	Prefrontal Cortex
